# Vascular composition data supporting the role of N-3 polyunsaturated fatty acids in the prevention of cardiovascular disease events

**DOI:** 10.1016/j.dib.2016.03.101

**Published:** 2016-04-07

**Authors:** Takayuki Ohwada, Tetsuro Yokokawa, Yuki Kanno, Yu Hotsuki, Takayuki Sakamoto, Kenichi Watanabe, Kazuhiko Nakazato, Yasuchika Takeishi

**Affiliations:** a Fukushima Red Cross Hospital, Department of Cardiology, Fukushima, Japan; b Fukushima Medical University, Department of Cardiology and Hematology, Fukushima, Japan

**Keywords:** N-3 polyunsaturated fatty acids, Eicosapentaenoic acid, Acute coronary syndrome, Stable coronary disease, Virtual histology-intravascular ultrasound

## Abstract

N-3 polyunsaturated fatty acids (PUFAs) are thought to have protective effects against cardiovascular disease. Here, we report the relationship between serum PUFA concentrations and plaque composition, as evaluated by virtual histology-intravascular ultrasound (VH-IVUS). Consecutive patients (*n*=61) who underwent percutaneous coronary intervention (PCI) were pre-operatively examined using VH-IVUS to assess the composition of culprit plaques. Gray-scale IVUS and VH-IVUS data of fibrous, fibro-fatty, necrotic core, and dense calcium regions of plaques were estimated at the minimal luminal area sites of culprit lesions. Serum levels of high-sensitivity C-reactive protein (hsCRP) and PUFAs, including eicosapentaenoic acid (EPA), docosahexaenoic acid (DHA), and arachidonic acid (AA), were compared between patients with (ACS, *n*=27) and without acute coronary syndrome (non-ACS, *n*=34) before PCI. Multiple logistic regression analysis of the data showed that EPA/AA under the median was more highly associated with ACS than hsCRP over the median. In addition, EPA/AA was negatively correlated with the percentage of fibrous plaque regions and EPA/AA and DHA/AA were positively correlated with the percentage of dense calcium regions in plaques. Furthermore, the correlation index of EPA/AA was the most highly (*R*=0.513) correlated with the percentage of dense calcium regions in plaques.

Specifications Table

**Table:** 

Subject area	Cardiology, Atherosclerosis
More specific subject area	Acute coronary syndrome, Poly-unsaturated fatty acids (PUFAs)
Type of data	Tables, Figures
How data was acquired	Prospective study
Data format	Raw data, Analyzed
Experimental factors	Virtual histology intravascular ultrasound images were quantified using echoPlaque 4.0 software (INDEC Systems, Inc.)
Experimental features	Correlations between VH-IVUS image data and serum PUFA levels in coronary plaques of 61 cardiovascular patients
Data source location	Fukushima, Japan
Data accessibility	Data is with this article

Value of the data

• The data provides information on the effect of PUFAs on the coronary vascular composition of cardiovascular disease patients.
• This data is valuable for further clinical examination of PUFAs and experimental vascular models.
• The analysis of plaque composition based on the gray-scale IVUS and VH-IVUS data is expected to be useful for assessing how PUFAs or other substances impact coronary vascular composition.
• The data may be potentially valuable to other researchers examining the relationship between PUFAs and vascular calcification.

## 1 Data

**Fig. 1:**
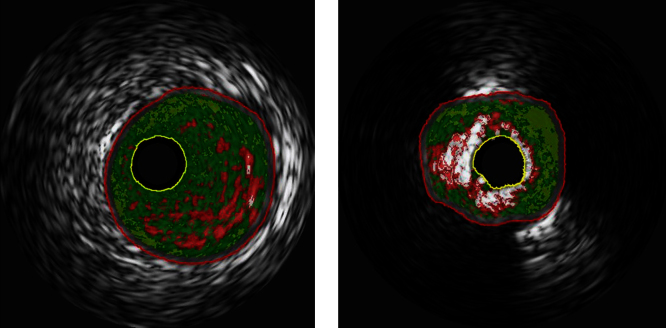
Representative VH-IVUS images of lesions with low (0.29; left panel) and high EPA/AA (0.82) ratios (right panel). Both images indicate dark green (fibrous), light green (fibro-fatty), red (necrotic core), and white (dense calcium). The low EPA/AA lesion (left panel) was predominantly fibrous (dark green, 66.89%) and contained few regions of dense calcium (white, 0.37%). In contrast, the high EPA/AA lesion (right panel) contained a high proportion of dense calcium regions (white, 22.76%).

**Fig. 2:**
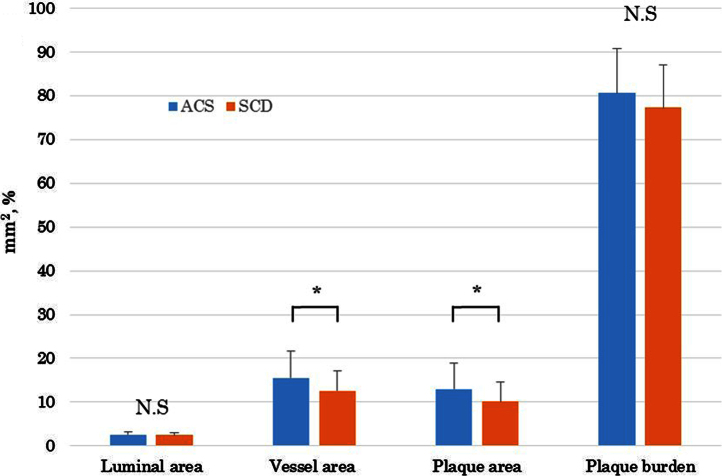
Comparisons of gray scale IVUS data between ACS and SCD patients. Lumina area, vessel area and plaque area are presented as area (mm^2^), and plaque burden is presented as percentage (%). N.S: not significant, *P<0.05.

**Fig. 3:**
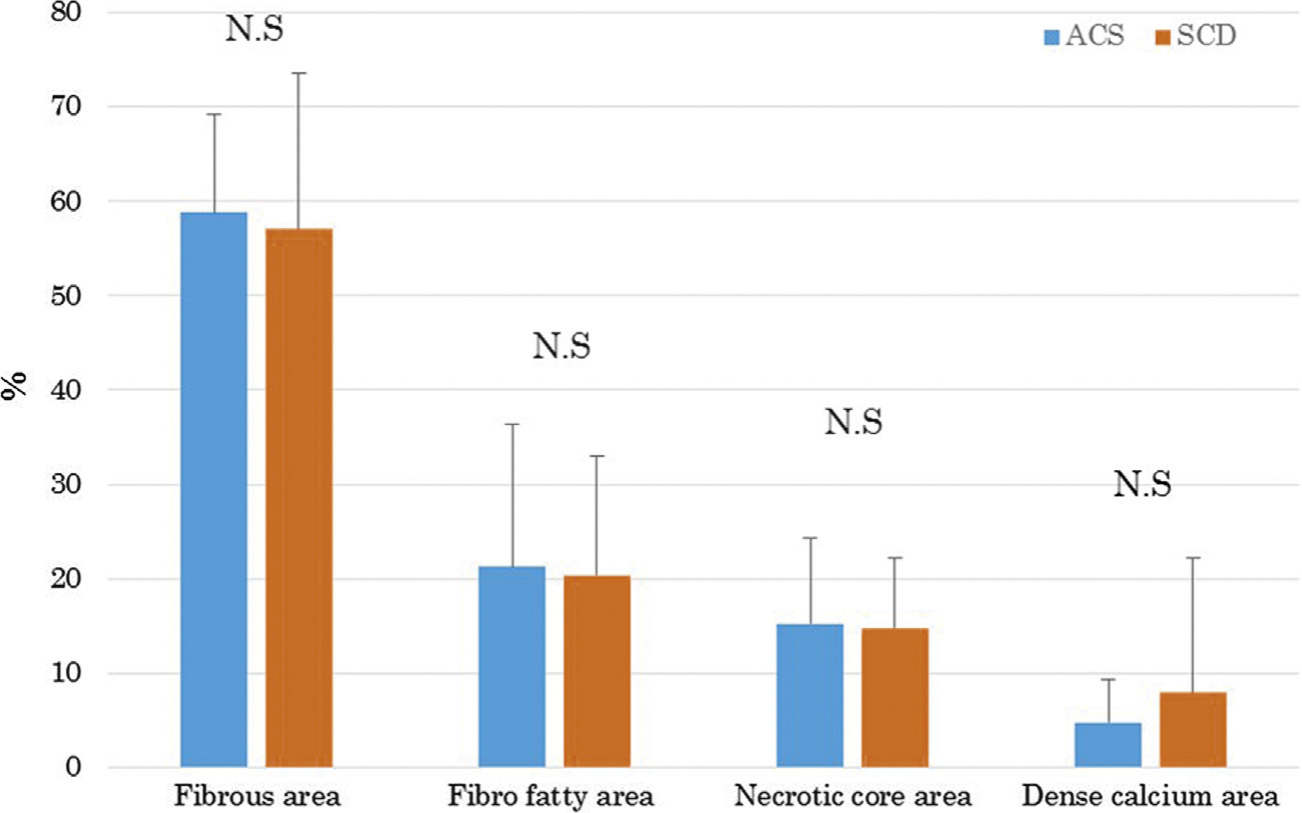
Comparisons of the percentages of the four examined component areas within plaque areas determined by VH-IVUS between ACS and SCD patients. N.S., not significant.

**Fig. 4:**
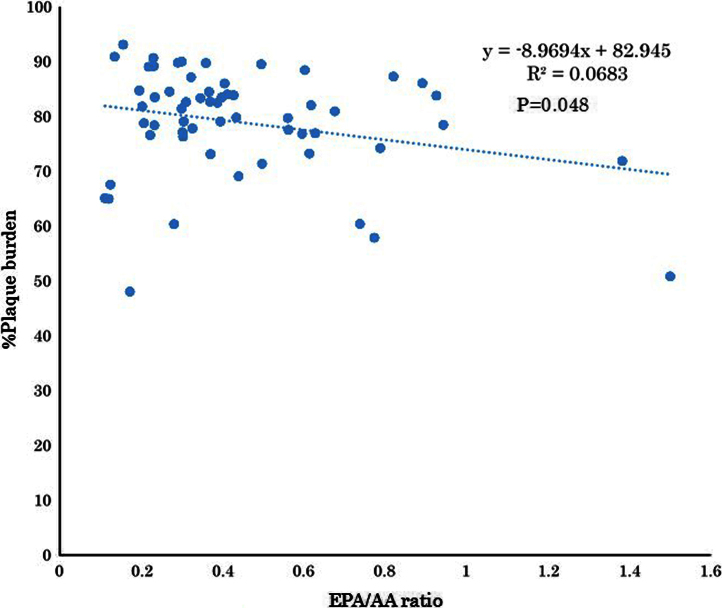
Correlation between EPA/AA and %Plaque burden. EPA/AA was inversely and significantly correlated with %Plaque burden (*R*=−0.261, *P*=0.048). This figure is based on the data presented in Table 5.

**Fig. 5:**
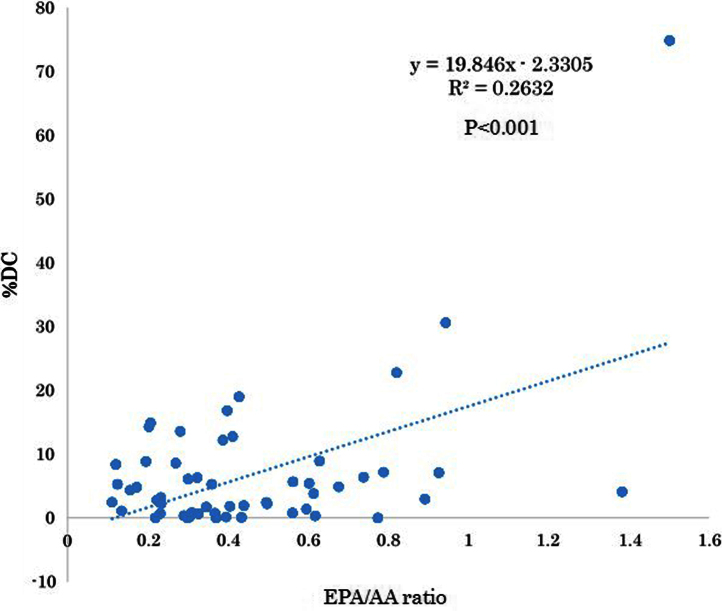
Correlation between EPA/AA and %DC. EPA/AA was significantly correlated with %DC (*R*=0.513, *P*<0.01). This figure is based on the data presented in Table 5.

**Fig. 6:**
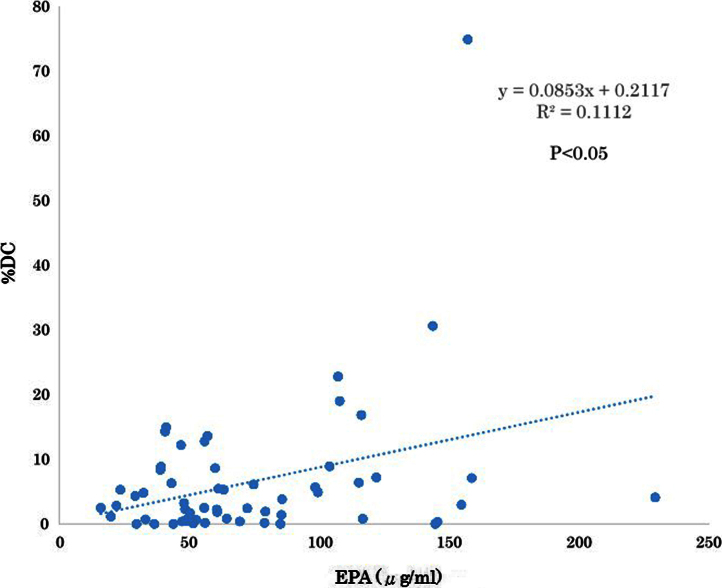
Correlation between EPA and %DC. EPA was significantly correlated with %DC (*R*=0.334, *P*=0.01). This figure is based on the data presented in Table 5.

**Fig. 7:**
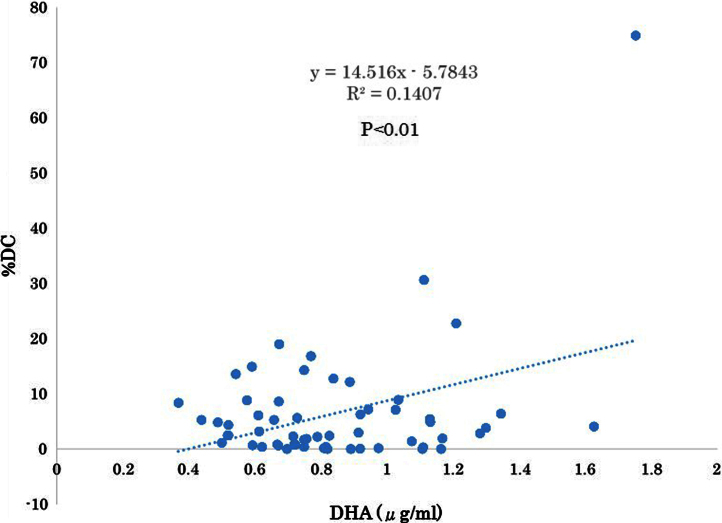
Correlation between DHA/AA and %DC. DHA/AA was significantly correlated with %DC (*R*=0.375, *P*=0.004). This figure is based on the data presented in Table 5.

The serum levels of eicosapentaenoic acid (EPA), EPA/arachidonic acid (AA), docosahexaenoic acid (DHA), and DHA/AA in acute coronary syndrome (ACS) patients were lower than those in stable coronary disease (SCD) patients (Table 1) (Fig. 1). In addition, the vessel and plaque areas in ACS patients were larger than those in SCD patients (Fig. 2); however, the percentage of fibrous, fibro-fatty, necrotic core, and dense calcium regions at sites of minimal luminal area within culprit lesions were comparable between the two patient groups (Table 2, Fig. 3), although the percentage of dense calcium regions in plaques tended to be higher in SCD patients than ACS patients (Table 2, Fig. 3). Univariate and multiple logistic regression analyses revealed that low DHA, low EPA, low DHA/AA, and low EPA/AA were independent factors for the risk of ACS (Table 3, Table 4). The percentage of dense calcium regions in plaques positively correlated with EPA/AA (Table 5, Fig. 5), EPA (Table 5, Fig. 6), and DHA (Table 5, Fig. 7), and EPA/AA was also inversely correlated with %plaque burden (ratio of plaque area to vessel area) (Table 5, Fig. 4).

## 2 Experimental design, materials and methods

### 2.1 Study population

This study was approved by the Fukushima Red Cross Hospital Ethics Committee (April 24, 2012), and written informed consent was obtained from all study subjects before enrollment. The study population consisted of 27 ACS patients, which included those with unstable angina (*n*=6) and acute myocardial infarction (AMI, *n*=21) and 34 SCD patients, which included those with stable effort angina pectoris (EAP, *n*=29) and silent ischemia (*n*=5). The diagnostic and exclusion criteria are described elsewhere [1], [2], [3]. The clinical histories of patients are listed in Table 1.

### 2.2 IVUS image acquisition and analysis

Intravascular ultrasound was used to examine culprit lesions. A phased-array, 20-MHz, 3.2-F IVUS catheter (Eagle Eye, Volcano Corp., Rancho Cordova, CA) was placed into the distal coronary artery and pulled back to the aorto-ostial junction using a motorized catheter pull-back system set at 0.5 mm/s (Eagle Eye, Volcano Corp.). The gray-scale IVUS and captured radiofrequency (VH-IVUS) data were analyzed using echoPlaque 4.0 software (INDEC Systems, Inc., City, State). Corresponding images of IVUS examinations were identified at culprit lesions between segments. The gray-scale IVUS and VH-IVUS images were analyzed at sites of minimal luminal area within culprit lesions, as previously described [4]. Gray-scale IVUS analysis was performed according to the American College of Cardiology Clinical Expert Consensus Document on Standards for Acquisition, Measurement and Reporting of Intravascular Ultrasound Studies [5]. In the conventional gray-scale IVUS analysis, cross-sectional images were quantified for luminal diameter, vessel diameter, intima diameter, luminal area, vessel area and plaque area. Plaque burden was calculated as the ratio of plaque area to vessel area. The remodeling index was calculated as the ratio of vessel area at the site of the measured lesion (sites of minimal luminal area) to the reference vessel area (average of the proximal and distal reference segments). The four VH-IVUS plaque components were color-coded as follows: dark green (fibrous), light green (fibro fatty), red (necrotic core), and white (dense calcium), and are reported as the area or percentage of plaque area. Representative images of lesions from ACS (low EPA/AA ratio; 0.29) and SCD patients (high EPA/AA ratio; 0.82) are shown in Fig. 1. The intraobserver (*r*=0.98, 0.98 and 0.99) and interobserver (*r*=0.96, 0.97, and 0.98) variability for the measurements were determined to be acceptable (Table 2).

### 2.3 Laboratory analyses

Blood examinations for lipid levels were performed within 2 days prior to PCI. Fasting blood samples were collected and after centrifugation and prompt freezing at −80 °C, serum samples were shipped to SRL, Inc. (Tokyo, Japan) for the measurement of dihomo-gamma-linolenic acid (DGLA), EPA, DHA, and AA levels using a gas chromatography method [6]. Plasma concentrations of hsCRP were quantified using the nephelometry method [7].

### 2.4 Statistical analysis

Data are presented as the mean±standard deviation. Categorical data are presented as number (*n*) and percentage (%). Categorical and continuous variables were compared between the ACS and SCD groups using the chi-square test and Student׳s unpaired *t*-test, respectively (Table 1, Table 2, Fig. 2, Fig. 3). Risk factors of ACS were identified using univariable and multivariable logistic regression analysis. All continuous data were categorized because linearity on the logit-scale could not be achieved with continuous covariables. The factors showing *P*<0.2 in the univariable test were entered into a multivariable logistic analysis. Significant prognostic factors were selected with a forward selection strategy using the likelihood ratio statistic (Table 3). In addition, multiple logistic analyses were performed using two models to avoid multicollinearity (Table 4). All statistical assessments were two-sided and evaluated at the 0.05 level of significance. Correlations of the EPA, EPA/AA, DHA, and DHA/AA data between the gray-scale and VH-IVUS data are shown in Table 5 and Fig. 4, Fig. 5, Fig. 6, Fig. 7. All statistical analyses were performed using SPSS 19.0 for Windows (SPSS Inc., Chicago, IL).

### 2.5 Limitations

Two limitations of this data warrant mention. First, the data was obtained at a single center from a relatively small number of patients. Data from a larger number of patients and multiple centers are needed to verify the correlations detected here. Second, the relationship between plaque calcification and PUFAs could not be determined from the data. Further experimental examination will be needed to determine the factors underlying this relationship.

## Competing interests

The authors declare that they have no competing interests.

## Figures and Tables

**Table 1:** Comparisons of patient characteristics and laboratory data between ACS and SCD patients.

Characteristic	ACS *n*=27 (%)	SCD *n*=34 (%)	*P*
Age (yrs±SD)	69±14	71±10	0.59511
Male sex	20 (74.1)	24 (70.6)	0.763
***Clinical histories***			
Diabetes mellitus	18 (66.7)	20 (58.8)	0.5301
Hypertension	19 (70.4)	24 (70.6)	0.9852
Dyslipidemia	17 (63.0)	13 (38.2)	0.055
Smoking	15 (55.6)	22 (64.7)	0.4674
Previous PCI	1 (3.7)	12 (35.3)	0.0028*
Previous CABG	0 (0)	1 (2.9)	0.3689
Hemodialysis	1 (3.7)	0 (0)	0.2579
***Oral medications***			
CCB	8 (29.6)	14 (41.2)	0.3509
ACEI/ARB	8 (29.6)	17 (50)	0.1081
Beta-blocker	3 (11.1)	1 (2.9)	0.2004
Diuretics	3 (11.1)	2 (5.9)	0.4596
Antiplatelet drugs	2 (7.4)	20 (58.8)	0.000033*
Statin	5 (18.5)	16 (47.1)	0.0198*
DT for DM	7 (25.9)	12 (35.3)	0.4326
***Culprit vessel***			
RCA	12 (44.4)	11 (32.4)	0.3785
LAD	13 (48.1)	17 (50.0)	0.8857
LCX	2 (7.4)	6 (17.6)	0.4266
***Laboratory data***			
TC (mg/dl)	203.1±46.6	181.4±30.1	0.0514
TG (mg/dl)	150.3±79.7	160.8±103.6	0.75
HDL-C (mg/dl)	51.6±16.2	50.6±12.3	0.8447
LDL-C (mg/dl)	131.8±42.3	111.4±27.5	0.1137
Creatinine (mg/dl)	1.2±0.9	0.9±0.3	0.1488
Uric acid (mg/dl)	6.0±1.8	5.4±1.3	0.1834
Na (mEq/l)	139.0±3.3	141.6±2.0	0.0036*
K (mEq/l)	4.2±0.4	4.3±0.4	0.6478
CL (mEq/l)	102.2±3.7	106.0±2.5	0.0003*
HbA1c	6.2±0.8	6.4±1.1	0.5434
WBC (×10^3^/μl)	9.4±3.2	6.3±2.0	0.0006*
Hb (g/dl)	12.9±3.0	13.6±2.2	0.3977
Plt (×10^3^/μl)	204±77.4	202.4±43.6	0.9235
hsCRP (ng/ml)	35410±61327	1958±3818	0.0088*
DGLA (μg/ml)	34.5±12.3	37.8±12.2	0.2962
AA (μg/ml)	173.4±48.7	174.4±45.5	0.9306
EPA (μg/ml)	49.5±28.3	92.9±42.1	0.00001*
DHA (μg/ml)	121.8±37.7	157.2±38.6	0.0007*
EPA/AA	0.297±0.143	0.568±0.306	0.00005*
DHA/AA	0.732±0.225	0.945±0.290	0.0027*

Values are the mean±SD or number of patients or (percentage). *:The difference between ACS and SCD is statistically significant with *P*<0.05. AA, arachidonic acid; ACEI/ARB, angiotensin-converting enzyme inhibitor and angiotensin II receptor blocker; ACS, acute coronary syndrome; CABG, coronary artery bypass grafting; CCB, Calcium-channel blockers; DGLA, dihomo-gamma-linolenic acid; DHA, docosahexaenoic acid; DT for DM, drug therapy for diabetes mellitus; EPA, eicosapentaenoic acid; HDL-C, high-density lipoprotein cholesterol; hsCRP, high-sensitivity C-reactive protein; LAD, left anterior descending coronary artery; LCX, left circumflex coronary artery; LDL-C, low-density lipoprotein cholesterol; PCI, percutaneous coronary intervention; Plt, platelet; PUFA, polyunsaturated fatty acids; RCA, right coronary artery; SCD, stable coronary disease; TC, Total cholesterol; TG, Triglyceride; WBC, white blood cell.

**Table 2:** Comparisons of gray scale-IVUS and VH-IVUS data between ACS and SCD patients.

	ACS (*n*=27)	SCD (*n*=34)	*P*
Gray scale-IVUS data			
Max luminal diameter (mm)	2.078±0.778	1.952±0.281	0.4368
Min luminal diameter (mm)	1.635±0.195	1.610±0.173	0.6172
Average luminal diameter (mm)	1.776±0.239	1.762±0.183	0.8131
Luminal area (mm^2^)	2.531±0.695	2.477±0.526	0.7398
Max vessel diameter (mm)	4.672±0.907	4.247±0.760	0.0573
Min vessel diameter (mm)	3.976±1.003	3.595±0.766	0.1063
Average vessel diameter (mm)	4.340±0.944	3.932±0.749	0.0715
Vessel area (mm^2^)	15.526±6.140	12.614±4.521	0.0422*
Plaque area (mm^2^)	12.995±5.895	10.137±4.457	0.04*
Plaque burden (%)	80.685±10.011	77.542±9.589	0.2287
Max intima thickness (mm)	1.951±0.625	1.619±0.482	0.026*
Min intima thickness (mm)	0.595±0.355	0.510±0.379	0.3914
Average intima thickness (mm)	1.233±0.425	1.078±0.381	0.15
Remodeling index	1.040±0.425	0.842±0.277	0.0533
VH-IVUS data			
Fibrous area (mm^2^)	5.822±3.266	4.217±2.047	0.035*
Fibrous area (%)	58.743±10.429	57.004±16.610	0.6443
Fibrous fatty area (mm^2^)	2.529±2.539	1.843±2.239	0.2791
Fibrous fatty area (%)	21.271±15.085	20.352±12.647	0.8015
Necrotic core area (mm^2^)	1.421±1.149	1.064±0.720	0.1749
Necrotic core area (%)	15.213±9.152	14.724±7.557	0.8246
Dense calcium area (mm^2^)	0.511±0.681	0.449±0.632	0.7237
Dense calcium area (%)	4.765±4.504	7.921±14.289	0.284

Data is shown as mean±SD. *:The difference between ACS and SCD is statistically significant with *P*<0.05. IVUS, intravascular ultrasound; Max, maximum; Min, minimum; VH-IVUS, virtual histology-intravascular ultrasound; Remodeling index, calculated as the ratio of vessel area at the measured lesion (minimum lumen area site) to the reference vessel area (average of the proximal and distal reference segments).

**Table 3:** Univariate logistic regression analysis for acute coronary syndrome.

Variable	OR	Single variable 95% CI	*P*
Male	1.19	0.383–3.699	0.763
Age>75 years:median	0.825	0.300–2.270	0.71
LDL-C:median	1.68	0.405–6.962	0.474
hsCRP≥1350:median	6.984	2.193–22.247	0.001*
AA≥167.5:median	0.825	0.300–2.270	0.71
DHA<137.3:median	6.857	2.207–21.304	0.001*
EPA<60.6:median	14.3	4.083–50.078	<0.001*
DHA/AA<0.79:median	3.231	1.122–9.303	0.030*
EPA/AA<0.37:median	8.4	2.609–27.047	<0.001*

OR, odds ratio. *:The value of OR is statistically significant with *P*<0.05. AA, arachidonic acid; DHA, docosahexaenoic acid; EPA, eicosapentaenoic acid; hsCRP, high-sensitivity C-reactive protein; LDL-C low-density lipoprotein cholesterol.

**Table 4:** Multiple logistic regression analysis for acute coronary syndrome.

	Model 1		Model 2	
Variable	OR (95% CI)	*P*	OR (95% CI)	*P*
hsCRP≧1350:median	4.456 (1.161–17.106)	0.029*	5.48 (1.458–20.595)	0.012*
EPA<60.6:median	8.879 (1.776–44.395)	0.008*		
DHA<137.3:median	1.521 (0.305–7.596)	0.609		
EPA/AA<0.37:median			8.235 (1.436–47.227)	0.018*
DHA/AA<0.79:median			0.998 (0.168–5.950)	0.999

*:The value of OR is statistically significant with *P*<0.05. AA, arachidonic acid; CI, confidence interval; DHA, docosahexaenoic acid; EPA, eicosapentaenoic acid; hsCRP, high-sensitivity C-reactive protein; OR, odds ratio.

**Table 5:** Correlation of EPA, EPA/AA, DHA and DHA/AA to gray-scale data and VH-IVUS data.

	EPA correlation index	*P*	EPA /AA correlation index	*P*	DHA correlation index	*P*	DHA/AA correlation index	*P*
Gray scale-IVUS data								
Max luminal diameter (mm)	−0.155	0.247	−0.137	0.304	−105	0.435	−0.047	0.724
Min luminal diameter (mm)	−0.143	0.264	−0.045	0.737	−0.07	0.603	0.158	0.237
Average luminal diameter (mm)	−0.077	0.567	−0.041	0.761	0.032	0.814	0.149	0.263
Luminal area (mm^2^)	−0.087	0.47	−0.057	0.673	0.009	0.944	0.141	0.293
Max vessel diameter (mm)	−0.296	0.024*	−0.321	0.014*	−0.233	0.079	−0.233	0.078
Min vessel diameter (mm)	−0.233	0.078	−0.253	0.055	−0.217	0.102	−0.201	0.129
Average vessel diameter (mm)	−0.267	0.043*	−0.285	0.030*	−0.222	0.093	−0.209	0.116
Vessel area (mm^2^)	−0.289	0.028*	−0.291	0.027*	−0.249	0.059	−0.213	0.109
Plaque area (mm^2^)	−0.286	0.029*	−0.293	0.025*	−0.258	0.051	−0.235	0.076
Plaque burden (%)	−0.185	0.163	−0.261	0.048*	−0.167	0.212	−0.253	0.055
Max intima thickness (mm)	−0.309	0.018*	−0.342	0.009*	−0.255	0.053	−0.28	0.033*
Min intima thickness (mm)	0.161	0.23	−0.182	0.175	−0.215	0.108	−0.233	0.082
Average intima thickness (mm)	−0.248	0.06	−0.283	0.031*	−0.2	0.132	−0.236	0.074
Remodeling index	−0.157	0.248	−0.131	0.341	−0.163	0.229	−0.083	0.541
VH-IVUS data								
Fibrous area (mm^2^)	−0.294	0.025*	−0.292	0.026*	−0.233	0.078	−0.214	0.106
Fibrous area (%)	−0.154	0.248	−0.264	0.046*	0.007	0.96	−0.155	0.245
Fibro fatty area (mm^2^)	−0.241	0.068	−0.249	0.06	−0.212	0.111	−0.179	0.179
Fibro fatty area (%)	−0.176	0.186	−0.229	0.084	−0.134	0.316	−0.147	0.271
Necrotic core area (mm^2^)	−0.073	0.584	−0.06	0.656	−0.161	0.228	−0.122	0.362
Necrotic core area (%)	0.107	0.424	0.141	0.292	−0.033	0.805	0.005	0.97
Dense calcium area (mm^2^)	0.121	0.367	0.125	0.35	0.036	0.789	0.026	0.847
Dense calcium (%)	0.334	0.011*	0.513	<0.001*	0.181	0.174	0.375	0.004*

*:The difference between ACS and SCD is statistically significant with *P*<0.05. IVUS, intravascular ultrasound; Max, maximum; Min, minimum.
